# Treatment patterns in patients with locally advanced and metastatic bladder cancer in Denmark 2015-2023 – an updated analysis

**DOI:** 10.2340/1651-226X.2025.43484

**Published:** 2025-05-07

**Authors:** Mette Nørgaard, Aurélie Mailhac, Karin Fagerlund, Torsten Strunz-McKendry, Mads Agerbæk, Jørgen Bjerggaard Jensen

**Affiliations:** a Department of Clinical Epidemiology, Arhus University Hospital and Department of Clinical Medicine, Aarhus University, Aarhus, Denmark; b Astellas Pharma A/S - Nordic Operation, Copenhagen, Denmark; c Astellas Pharma Europe Ltd, Addlestone, UK; d Department of Oncology, Aarhus University Hospital and Department of Clinical Medicine, Aarhus University, Aarhus, Denmark; e Department of Urology, Aarhus University Hospital and Department of Clinical Medicine, Aarhus University, Aarhus, Denmark

**Keywords:** Bladder cancer treatment patterns, nationwide cohort study

## Introduction

Treatment patterns in locally advanced and metastatic urothelial bladder cancer (La/mUBC) are changing. Recently, consolidating immune-oncology (IO) therapy has been introduced for patients responding to platinum-based therapy. Yet, updated knowledge about routine use of these treatments remains scarce. We previously found that around 50% of La/mUBC patients in Denmark received systemic anti-cancer treatment [1]. In the present manuscript, we have included information from the recently established Danish National Hospital Medication Register (DHMR) [2] to further describe treatment patterns in Danish La/mUBC patients in a routine clinical care setting.

## Materials and methods

This was a nationwide registry-based cohort study. All Danish residents are provided tax-funded medical care, guaranteeing free access to hospitals [3]. The unique personal identifier, the Civil Registration Number, allowed data linkage at an individual level [4].

The study population included all patients with incident histologically verified La/mUBC registered in the period 2015–2023 in the Danish National Patient Registry (DNPR) [5] and in the Danish Pathology Register (DPR) [6, 7] and no previous cancer, as described previously [1]. We categorised the patients into those who presented with La/mUBC (de novo La/mUBC) and patients who progressed to La/mUBC from non-invasive or localised muscle-invasive bladder cancer.

From DNPR, we identified lines of treatment using a previously validated algorithm [8]. We also obtained information about systemic cancer treatment from the Danish National Hospital Medication Register [2]. According to the National Clinical Guidelines, carboplatin plus gemcitabine is the treatment of choice for cisplatin-ineligible patients. IO is also reimbursed for patients with PD-L1 positive tumors and is primarily used for those ineligible for combination chemotherapy. Chemotherapy was classified into platinum-based (cisplatin or carboplatin-based) and other. IO was classified by type into pembrolizumab, atezolizumab, nivolumab, and avelumab. We defined avelumab as maintenance treatment if started within 12 weeks after the last dose of first-line platinum-based chemotherapy.

We followed patients from the date of pathology-confirmed La/mUBC (index date) until death or last recorded follow-up (June 30, 2024). We computed the number of patients who received systemic anti-cancer therapy, the number of patients who received first-, second-, and third-line therapy, and specific treatment types. We constructed a Sankey plot to visualise treatment patterns in patients diagnosed in 2021–2023.

All statistical analyses were conducted using the SAS statistical software package, v. 9.4 (SAS Institute, Cary, NC). This study was reported to the Danish Data Protection Agency through registration at Aarhus University (record number 2016‐051‐000001-718).

## Results

We identified 1,930 patients registered in the DPR and DNPR with La/mUBC and no previous cancer in the period 2015–2023. Of these, 324 (17%) had de novo La/mUBC while 1,606 had progressed to La/mUBC. Of the 1,930 patients, 71% were men, and the median age was 73 years. At study inclusion, 11% had a diagnosis of chronic obstructive pulmonary disease, 8% had a previous myocardial infarction, and 10% had diabetes.

Median follow-up in the entire cohort was 12 months (interquartile range [IQR] 5;29).

During follow-up, 1,016 of the 1,930 patients started first-line treatment (53%, 46% of all women and 55% of men), of these, 421 also started second-line treatment, and 149 started third-line treatment. Among patients initiating first-line treatment, 24% received IO therapy compared with 45% of those who started second-line therapy and 21% of those starting third-line therapy.

**Figure 1:**
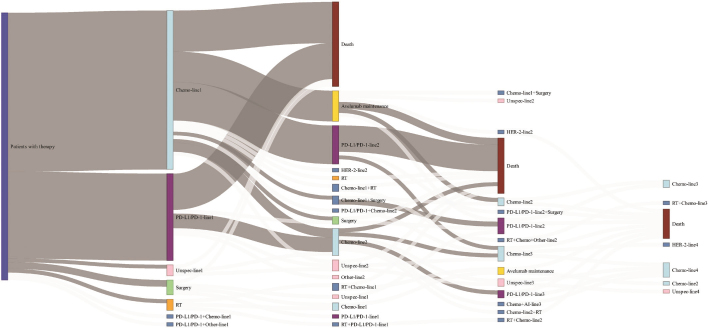
Sankey plot visualising treatment choices in 653 patients with La/mUBC diagnosed in the period 2021–2023. Due to low numbers, the thickness of the white lines does not reflect the actual number of patients in these lines. Accordingly, the categories with several white lines (e.g. surgery and radiotherapy) have been slightly inflated in this illustration.

Among 653 patients diagnosed in 2021–2023, 354 (54%) started first-line treatment (Figure 1). Of these, 231 (65%) started chemotherapy, whereof 86 (39%) were cisplatin-based, 108 (48%) carboplatin-based and 29 (13%) were other types. In addition, 123 (35%) started IO, the majority (74%) with Atezolizumab. Among the 194 patients who received platinum-based chemotherapy, 48 patients (25%) also started avelumab maintenance treatment. During follow-up, 125 started second-line treatment, of whom 49% received chemotherapy (22% cisplatin-based, 34% carboplatin-based and 44% other types) and 51% received IO (89% of these with atezolizumab).

The median survival was lower in de novo La/mUBC (7 months [95% confidence interval, CI: 6–9]) compared with those who progressed to La/mUBC (15 months [95% CI: 14–17]), whereas it was 13 months (95% CI: 12–14) from index date in the entire cohort. From the date of first-line treatment, the median survival was 12 months (95% CI: 11–13), while it was 10 months (95% CI: 9–12) from the date of second-line treatment.

## Discussion

In this population-based cohort study, describing treatment patterns in La/mUBC treated in routine clinical care, we included information from the recently established Danish National Hospital Medication Register which allowed us to obtain more granulated treatment data. More than half of the patients diagnosed with La/mUBC received systemic treatment. This was within the range reported in previous studies [9, 10] and comparable to the findings by Swami et al., who found that in multiple large cohort studies, almost half of the patients did not receive any treatment for metastatic disease, while only around 15%–20% receive second-line therapy [11]. Our finding that one quarter of those who received platinum-based chemotherapy, also received avelumab maintenance treatment was very similar to the data reported in Germany [12] and Spain [13] , whereas a recent French study reported avelumab use in about one fifth of patients starting first-line chemotherapy [14].

We used data from Danish health registries which are prospectively collected and have virtually complete follow-up. We used an algorithm developed for the Danish Bladder Cancer Database [15] to identify bladder cancer patients. However, since we required a pathologically confirmed diagnosis, we may have missed patients with diagnoses confirmed by imaging only. We also lacked information on clinical progression following diagnosis of La/mUBC, and therefore, we could not identify those who weren’t candidates for maintenance treatment following first-line treatment due to disease progression. Another weakness of observational data was our lack of data on lifestyle factors such as smoking which would contribute to a better characterisation of our study populations.

In conclusion, patients with La/mUBC have a poor prognosis, and in routine clinical care, only around half of the patients received systemic anti-cancer treatment, which was mostly chemotherapy. The Danish medical databases seem to be a useful resource to describe treatment patterns, which is further strengthened by the inclusion of data from the Danish National Hospital Medication Register.

## Data Availability

The data for this study are placed and were accessed on a secured server at the Danish Health Data Authority. Data are not publicly available due to Danish legislation.
